# Impact of Health Service Quality on Patient Loyalty in Government and Private Hospitals in the Asir Region, Saudi Arabia

**DOI:** 10.7759/cureus.100963

**Published:** 2026-01-06

**Authors:** Sadeem M Bin Libdah, Mohamed A Alqurashi

**Affiliations:** 1 Health Administration, Faculty of Economics and Administration, King Abdulaziz University, Jeddah, SAU; 2 Health Law and Policy, King Abdulaziz University, Jeddah, SAU

**Keywords:** asir region, empathy, health service quality, patient loyalty, responsiveness, saudi arabia

## Abstract

Background

Healthcare service quality is a critical determinant of patient satisfaction and loyalty. In Saudi Arabia, several hospitals continue to face service-related challenges, including prolonged waiting times, inconsistent communication, and limited patient-centered practices. This study aimed to examine the impact of the five dimensions of healthcare service quality on patient loyalty in government and private hospitals with 100 or more beds in the Asir region.

Methods

A quantitative cross-sectional study was conducted between April and October 2024 using a self-administered bilingual (Arabic-English) survey disseminated online. A total of 1,043 valid responses were obtained. Service quality and loyalty were measured using a five-point Likert scale. Descriptive statistics, Pearson correlation, and hierarchical cluster analysis were carried out using the Statistical Package for the Social Sciences (SPSS) software version 23 (IBM Corp., Armonk, USA).

Results

A total of 1,043 respondents (100%) were included in the analysis. Participants were predominantly aged 30-49 years (n=581; 55.7%) and female (n=559; 53.6%). Asir Central Hospital was the most frequently visited facility (n=461; 44.2%). Pearson correlation analysis showed that all five healthcare service quality dimensions were significantly and positively associated with patient loyalty (p < 0.001). Responsiveness demonstrated the strongest correlation with loyalty (r = 0.480), followed by empathy (r = 0.478), assurance (r = 0.463), reliability (r = 0.449), and tangibility (r = 0.440). Hierarchical cluster analysis identified three loyalty groups: high loyalty (n=563; 54.0%), average loyalty (n=378; 36.2%), and low loyalty (n=102; 9.8%).

Conclusion

Healthcare service quality strongly and significantly influences patient loyalty in the Asir region. Enhancing responsiveness, empathy, and assurance should be prioritized to strengthen patient trust and retention. Improving service quality is essential for building a sustainable, patient-centered healthcare system.

## Introduction

The quality of healthcare services has become a central focus of health systems worldwide, particularly as patient expectations rise and health organizations face increasing competitive and operational pressures [1,2]. Healthcare is a unique sector within the broader service industry due to its complexity, high emotional stakes, and its direct connection to human well-being. As service environments become more patient-centered, the concept of healthcare service quality has evolved to encompass clinical effectiveness, safety, communication, empathy, and the overall care experience [1]. Globally, healthcare systems are shifting from volume-based care to value-based models, emphasizing quality outcomes and patient satisfaction as core indicators of system performance [3,4].

In Saudi Arabia, healthcare transformation is a key pillar of Vision 2030, which seeks to enhance accessibility, efficiency, and quality across public and private sectors [5,6]. Despite significant investments, studies indicate that Saudi hospitals continue to face challenges such as long waiting times, inconsistent communication, and variations in service delivery [7,8]. These challenges may undermine patient trust and reduce the likelihood that individuals will return to the same facility, thereby weakening patient loyalty. Patient loyalty is increasingly recognized as a critical outcome for healthcare organizations because it reduces system costs, enhances continuity of care, and strengthens public trust [9].

Service quality is often operationalized through the Service Quality (SERVQUAL) dimensions, i.e, tangibility, reliability, responsiveness, assurance, and empathy, which collectively shape patients’ perceptions and behavioral intentions [10,11]. These dimensions have consistently been shown to influence patient satisfaction, trust, and loyalty. However, the extent and magnitude of their impact vary across cultural, institutional, and regional contexts. In the Asir region, a mountainous and geographically dispersed area, healthcare utilization is shaped by unique demographic and infrastructural factors. Despite this, limited research has explored how patients in this region perceive the quality of healthcare services and how these perceptions influence loyalty.

This study aims to fill this research gap by examining the impact of healthcare service quality on patient loyalty in government and private hospitals in the Asir region. By analyzing the five key dimensions of service quality and measuring loyalty levels among hospital visitors, the study seeks to provide evidence-based insights that can support healthcare managers, policymakers, and practitioners in designing interventions that enhance patient experience and foster long-term loyalty.

## Materials and methods

Study design

This study employed a quantitative, cross-sectional research design to examine the impact of healthcare service quality on patient loyalty among visitors to government and private hospitals in the Asir region of Saudi Arabia. The study was conducted between April 19 and October 31, 2024, targeting adult patients who had received services in hospitals with a capacity of 100 beds or more.

Study settings and population

The participating hospitals were categorized into two distinct sectors (government hospitals and Private hospitals). Government hospitals (n = 13) included Asir Central Hospital, Abha Maternity and Children’s Hospital, Al-Khamis Maternity and Children’s Hospital, Mahayil General Hospital, Khamis Mushayt General Hospital, Mental Health Hospital in Abha, Sarat Abida General Hospital, Dhahran Al Janoub General Hospital, Al-Majardah General Hospital, Rijal Almaa General Hospital, Balasmar General Hospital, Al-Namas General Hospital, and Ahad Rufaida Hospital. Private hospitals (n = 4) included Saudi German Hospital, Hayat National Hospital, Abha Private Hospital, and Al Madawaa Specialized Hospital.

The Asir region has a population of more than 2 million residents, dispersed across urban, semi-urban, and rural settings. Because of this geographic spread, collecting data in person from multiple hospitals posed logistical challenges. To overcome this, an online self-administered questionnaire was used.

Sampling

The sample size was determined using the Raosoft Sample Size Calculator (Raosoft Inc., Seattle, USA) with a 99% confidence interval, a 4% margin of error, and a population size of 2,024,285. The minimum required sample size was calculated as 1,037 respondents. A total of 1,183 responses were received, of which 140 were excluded because respondents indicated they had not received services from any Asir hospital. The final sample consisted of 1,043 valid responses, exceeding the minimum requirement and strengthening the study’s generalizability.

Inclusion and exclusion criteria

Participants were eligible for inclusion if they were 18 years or older, had received healthcare services from one of the targeted hospitals in the Asir region, and expressed willingness to participate in the study. This was followed by the provision of informed consent prior to completing the questionnaire. Participants were excluded if they were under 18 years of age, did not express willingness to participate, did not provide informed consent, or had not received services from a qualifying hospital.

Data collection instrument

The questionnaire used in this study was adapted from a previously published and validated instrument. Specifically, it was based on the SERVQUAL-derived questionnaire developed by Amor et al. [8]. The original tool was modified and translated into Arabic to ensure cultural relevance, linguistic clarity, and appropriateness for the Saudi healthcare context. The final questionnaire was structured into three distinct sections.

Section 1 collected participants’ demographic and hospital-related information, including age, gender, and the hospital visited.

Section 2 assessed perceived healthcare service quality using 15 items covering the five SERVQUAL dimensions: tangibility, reliability, responsiveness, assurance, and empathy. Responses in this section were rated on a five-point Likert scale, ranging from 1 (strongly disagree) to 5 (strongly agree).

Section 3 evaluated patient loyalty through seven items, capturing behavioral intentions such as willingness to revisit the hospital, likelihood of recommending the facility to others (word-of-mouth), and responses to dissatisfaction or complaints.

Data collection procedure

The questionnaire was disseminated through WhatsApp groups with emphasis on encouraging group members to further share the survey link. No specific healthcare services were predefined, as the questionnaire was general and aimed at assessing the performance of any healthcare provider within the hospital, whether in outpatient clinics or inpatient services.

Data analysis

Data were analyzed using SPSS version 23 (IBM, Inc., NY, USA). Analysis involved two major components: (1) Descriptive statistics were performed, and frequencies and percentages were calculated for demographic variables to understand the characteristics of the sample. (2) Inferential statistics involved Pearson correlation analysis, which examined the strength and significance of relationships between the five service quality dimensions and patient loyalty. The p-value was set at p<0.01 for statistical significance. Pearson correlation was chosen because the data met assumptions of normality and linearity. Hierarchical cluster analysis involved Ward’s Method, which was applied to classify respondents into three loyalty groups (high, average, and low loyalty) based on their responses to loyalty items. This provided a deeper understanding of loyalty patterns within the population.

Ethical considerations

Ethical approval was obtained from the Asir Institutional Review Board (REC: 3-4-2024). Participants were informed of their rights, including voluntary participation and the ability to withdraw at any time. Data were stored securely on a password-protected device and anonymized to ensure confidentiality.

## Results

A total of 1,043 respondents participated in the study, all of whom had received healthcare services at hospitals in the Asir region (including outpatient clinics and inpatient care) with a capacity of at least 100 beds. 

Participants’ characteristics

Participants’ age distribution was presented in ascending age groups. Individuals aged 18-29 years accounted for 185 respondents (17.7%), followed by those aged 30-39 years with 291 respondents (27.9%). Participants aged 40-49 years comprised 290 respondents (27.8%), while those aged 50-59 years included 197 respondents (18.9%). The smallest proportion of respondents were aged 60 years or older, representing 80 individuals (7.7%). Overall, middle-aged adults constituted the majority of hospital service users during the study period.

With regard to gender, a slightly higher proportion of respondents were female (n=559; 53.6%), compared with male respondents (n=484; 46.4%) (Table 1). 

**Table 1:** Demographic and Hospital Visitation Characteristics of Respondents (N = 1,043)

Category	Sub-Category	n	%
Age Group	18–29 years	185	17.74%
	30–39 years	291	27.90%
	40–49 years	290	27.80%
	50–59 years	197	18.89%
	≥60 years	80	7.70%
Gender	Male	484	46.40%
	Female	559	53.60%
Hospital Visited	Asir Central Hospital	461	44.2%
	Abha Private Hospital	122	11.7%
	Abha Maternity & Children Hospital	110	10.5%
	Khamis Mushayt General Hospital	87	8.3%
	Saudi German Hospital	52	4.9%
	Mahayil General Hospital	47	4.5%
	Al-Khamis Maternity & Children Hospital	39	3.7%
	Rijal Alma General Hospital	30	2.9%
	Ahad Rafidah General Hospital	24	2.3%
	Al-Hayat National Hospital	23	2.2%
	Mental Health Hospital in Abha	11	1.2%
	Al-Namas General Hospital	12	1.2%
	Al-Madawaa Specialized Hospital	10	0.9%
	Sarat Ubaida General Hospital	6	0.6%
	Al-Majarda General Hospital	3	0.3%
	Balasmar General Hospital	3	0.3%
	Dhahran Al Janoub General Hospital	3	0.3%

Hospital visitation characteristics

Analysis of hospital visitation patterns indicated that Asir Central Hospital was the most frequently visited facility, reported by 461 respondents (44.2%). Other commonly visited hospitals included Abha Private Hospital (n=122; 11.7%), Abha Maternity and Children Hospital (n=110; 10.5%), and Khamis Mushayt General Hospital (n=87; 8.3%).

Hospitals with comparatively lower visitation rates included Saudi German Hospital (n=52; 4.9%), Mahayil General Hospital (n=47; 4.5%), and Al-Khamis Maternity and Children Hospital (n=39; 3.7%). Several facilities, including Al-Majardah General Hospital (n=3; 0.3%), Balasmar General Hospital (n=3; 0.3%), and Dhahran Al Janoub General Hospital (n=3; 0.3%), accounted for a minimal percentage of total visits (Table 1).

Correlation between service quality and loyalty

Pearson correlation analysis revealed significant positive correlations between all five dimensions of healthcare service quality and patient loyalty (p < 0.01) (Table 2). The strength of these correlations varied. Responsiveness (r = 0.480) was the strongest predictor of loyalty. Empathy (r = 0.478) also demonstrated a strong correlation, highlighting the importance of personalized attention, respect, and emotional support. Assurance (r = 0.463) was similarly influential, while reliability (r = 0.449) and tangibility (r = 0.440) showed moderate but significant correlations.

**Table 2:** Correlation Between Service Quality Dimensions and Patient Loyalty *Pearson correlation coefficients. Significant at p < 0.05; all reported correlations were significant at p < 0.001.

Service Quality Dimension	Pearson Correlation (r)	Significance (p-value)
Tangibility	0.440	< 0.001*
Reliability	0.449	< 0.001*
Responsiveness	0.480	< 0.001*
Assurance	0.463	< 0.001*
Empathy	0.478	< 0.001*

Cluster analysis of loyalty levels

Hierarchical cluster analysis classified respondents into three distinct loyalty groups based on their responses to loyalty-related items. The high-loyalty group included 563 respondents (54.0%), representing more than half of the study population and indicating strong intentions to revisit and recommend the hospital.

The average-loyalty group comprised 378 respondents (36.2%), reflecting moderate levels of loyalty behaviors. The low-loyalty group included 102 respondents (9.8%), representing a smaller subset of participants who reported weaker intentions to return or recommend the healthcare facility. Table 3 presents the results of a hierarchical cluster analysis, classifying respondents into three distinct loyalty clustersbased on their responses.

**Table 3:** Loyalty Cluster Groups (Based on Hierarchical Cluster Analysis)

Level of loyalty	N	%
High loyalty	563	54.0%
Average loyalty	378	36.2%
Low loyalty	102	9.8%

## Discussion

The aim of this study was to assess the impact of healthcare service quality on patient loyalty in government and private hospitals in the Asir region. The findings provide important insights into how various service quality dimensions influence loyalty and illuminate opportunities for strategic improvement in the Saudi healthcare system.

It is important to note that the Asir region hosts a multi-ethnic population, including Saudi nationals and expatriate communities from diverse cultural and linguistic backgrounds. Although ethnicity was not analyzed as an independent variable in this study, the observed perceptions of service quality and patient loyalty likely reflect this heterogeneity. Cultural norms, language proficiency, prior healthcare experiences, and expectations of care may influence how patients interpret responsiveness, empathy, and assurance [12]. Therefore, the findings should be understood as representing aggregated perceptions across a diverse patient population rather than ethnicity-specific experiences.

Responsiveness emerged as the strongest correlate of patient loyalty (r = 0.480), suggesting that when patients perceive staff as prompt and willing to assist, they are more likely to remain loyal. This supports previous findings suggesting that delays in communication, assistance, and service provision are major drivers of patient dissatisfaction [13,14]. In Saudi Arabia, waiting times and service delays have historically been reported as challenges in public hospitals [15]. The strong correlation in this study underscores the importance of timely care, as patients who feel their needs are acknowledged promptly are significantly more likely to return and recommend the hospital.

Differences between government and private hospitals are frequently noted in Saudi patients’ experiences and may shape perceived service quality and loyalty. Evidence comparing sectors in Saudi Arabia suggests that private hospitals are often rated higher on perceived service quality dimensions (particularly assurance and responsiveness), potentially reflecting shorter queues, more flexible appointment systems, and higher perceived service orientation [16,17]. In contrast, government (Ministry of Health and other public) hospitals are commonly valued for broader access, large catchment coverage, and comprehensive services, but patient experience challenges are often linked to longer waiting times and operational congestion, which can negatively influence satisfaction and subsequent loyalty behaviors [15,18,19].

These sectoral patterns may help interpret why relational and process-oriented SERVQUAL dimensions (e.g., responsiveness and empathy) showed strong associations with loyalty in the present study: when patients experience delays or limited staff time, more commonly reported in higher-volume public settings, perceptions of responsiveness and individualized attention may decline, with downstream effects on loyalty [16,17]. On the other hand, when patients perceive faster responses and stronger communication, frequently reported in private settings, loyalty intentions may increase [16,17,20].

Importantly, Saudi literature also indicates that sector differences are not absolute and may vary by region, facility type (e.g., tertiary referral hospitals), and patient expectations [21,22]. Therefore, our findings should be interpreted as reflecting aggregated experiences across both sectors in the Asir region rather than implying uniform superiority of one sector across all dimensions

Empathy was the second strongest predictor of loyalty (r = 0.478), highlighting the importance of personalized attention, respect, and emotional support. In healthcare, empathy encompasses understanding patients' concerns, providing personalized attention, and demonstrating compassion. This finding aligns with a previous study that established empathy as a significant determinant of loyalty [23]. In Asir, where cultural expectations emphasize respect, dignity, and personal relationships, empathy plays a central role in shaping the patient experience. The results suggest that healthcare providers must cultivate strong interpersonal skills to build meaningful, trust-based relationships.

Assurance showed a strong correlation with loyalty (r = 0.463), indicating that patients value feeling safe, confident, and assured by healthcare professionals. Assurance relates to the confidence patients have in the knowledge, credibility, and professionalism of healthcare staff. In the context of Saudi Arabia, where patients increasingly seek high-quality, specialized care, assurance is crucial. The finding reinforces previous studies that emphasize trust as a core component of loyalty [24,25]. When patients feel safe and believe in the competence of their providers, they are more likely to continue seeking care from the same hospital [26].

Although reliability (r = 0.449) and tangibility (r = 0.440) demonstrated lower correlations, they remain significant contributors to loyalty, demonstrating that consistent service delivery and well-maintained facilities remain essential, albeit slightly less influential compared to relational aspects of care. Reliability relates to consistency of service delivery and meeting expectations, while tangibility reflects the physical aspects of care, including cleanliness, equipment, and hospital environment. These findings align with those of Doss et al. [27] and Amor et al. [8], indicating that while relational factors are becoming increasingly important, foundational aspects such as physical infrastructure and consistent service remain essential. 

The identified significant positive correlations between all five dimensions of healthcare service quality and patient loyalty support the underlying SERVQUAL model, suggesting that both functional and technical components of care influence patient loyalty in the Asir region.

This study reinforces global evidence that service quality is a multidimensional construct influencing loyalty through various pathways. Consistent with Liu et al. [28], the findings suggest that patient trust mediates the relationship between service quality and loyalty. In hospitals, trust was shown to enhance loyalty even when service quality was moderate [9,28], a pattern that also seems applicable in the Asir region.

Saudi-specific literature indicates mixed performance across hospitals. Mughal [29] found that public hospitals sometimes outperform private ones in perceived satisfaction, whereas Alluhaymid et al. [30] noted deficiencies in all five quality dimensions in public facilities. This suggests regional variations, underscoring the importance of localized research like the present study.

The finding that 54% of respondents exhibited high loyalty is encouraging and indicative of strong patient engagement. However, the 36.2% average and 9.8% low loyalty groups reveal significant room for improvement. These groups likely include individuals who experienced inconsistent communication, delays, or a lack of personalized attention. Loyalty is strongly tied to the sustainability of healthcare facilities [31]. Moreover, loyal patients reduce the cost of recruitment, promote positive word-of-mouth, and support continuity of care [9]. Therefore, for Asir hospitals, enhancing loyalty is essential to improving patient outcomes, optimizing resource allocation, and strengthening institutional reputation.

The findings of this study highlight several practical implications for healthcare managers and policymakers seeking to improve service quality and strengthen patient loyalty in hospitals across the Asir region. First, improving responsiveness requires hospitals to streamline administrative workflows, eliminate bottlenecks, and adopt digital health innovations. Tools such as electronic queue systems, online check-ins, and mobile appointment applications have been shown internationally to reduce waiting times and enhance the overall patient experience [32-34]. Implementing these systems can directly address one of the strongest predictors of loyalty identified in this study.

While empathy may be partly innate, it can be strengthened through targeted professional development. Structured training in patient-centered communication, emotional intelligence, and cultural sensitivity should therefore be integrated into continuous education programs for healthcare staff [35]. Such training ensures that healthcare workers are equipped to meet patients’ emotional and informational needs consistently.

Another essential implication relates to trust and professionalism. Enhancing assurance requires visible demonstrations of competence, which can be achieved through ongoing professional development, clinical certification, and adherence to evidence-based practice. Investment in advanced medical technologies, continuous training, and strong clinical governance structures could reinforce patient confidence in the reliability and safety of healthcare services [35-37]. 

Finally, improvements to the physical environment are also necessary. Clean, well-maintained facilities, modern equipment, and comfortable waiting areas have a strong influence on patients’ perceptions of service quality [38]. These enhancements are especially important in competitive private-sector hospitals, where physical surroundings often shape initial impressions and overall satisfaction. Therefore, upgrading facility aesthetics and ensuring a welcoming environment can significantly strengthen patient loyalty.

By demonstrating that all five service quality dimensions significantly predict patient loyalty in a Saudi Arabian context, this study reinforces the robustness and applicability of SERVQUAL in Middle Eastern healthcare settings. While global studies have confirmed these relationships across various countries and health systems [39-41], the present findings add important regional specificity. In particular, the results highlight the especially strong influence of relational dimensions such as empathy and responsiveness, which appear more culturally salient in Saudi society. This suggests that in collectivist and high-context cultures, interpersonal aspects of care may weigh more heavily in shaping loyalty than purely technical or infrastructural components. This study, therefore, expands our understanding of how cultural context moderates the relationship between service quality and patient behavior, reinforcing the need for culturally informed quality improvement strategies.

Despite its strengths, this study has several limitations that should be considered when interpreting the findings. First, the cross-sectional study design precludes causal inference and limits conclusions to associations between healthcare service quality and patient loyalty. Second, data were collected through an online, self-administered survey, which may introduce selection bias by favoring individuals with internet access and higher digital literacy, as well as recall and social desirability biases. Third, although the study included both government and private hospitals, responses were analyzed in aggregate and were not stratified by hospital sector. This lack of differentiation limits the ability to directly compare service quality perceptions and loyalty between public and private healthcare settings. The reliance on self-reported measures also may not fully capture objective service quality or actual utilization behaviors. Future studies employing mixed-methods designs, longitudinal approaches, and sector-specific analyses are warranted to address these limitations and provide a more nuanced understanding of patient experiences across healthcare settings. Additionally, the study did not stratify analyses by ethnic background, which limits the ability to explore potential differences in service quality perceptions and loyalty across ethnic groups within the region. Thus, future studies should explicitly incorporate ethnicity and language preference as analytical variables to better understand how cultural diversity shapes patient expectations, experiences, and loyalty within Saudi Arabia’s increasingly multicultural healthcare system. 

## Conclusions

This study demonstrates that healthcare service quality significantly influences patient loyalty in Asir region hospitals. All SERVQUAL dimensions (tangibility, reliability, responsiveness, assurance, and empathy) showed strong positive associations with loyalty, with responsiveness and empathy emerging as the most influential predictors. These findings underscore the significance of timely care, compassionate communication, and professional competence in influencing patients’ behavioral intentions. Although most respondents reported high loyalty, substantial opportunities remain to improve patient experience through workflow optimization, staff development, and facility enhancements. Strengthening service quality is essential for advancing patient-centered care and supporting Saudi Arabia’s ongoing healthcare transformation under Vision 2030.
